# LC-MS based simultaneous profiling of adrenal hormones of steroids, catecholamines, and metanephrines

**DOI:** 10.1016/j.jlr.2023.100453

**Published:** 2023-10-06

**Authors:** Jongsung Noh, Chaelin Lee, Jung Hee Kim, Seung Woon Myung, Man Ho Choi

**Affiliations:** 1 Center for Advanced Biomolecular Recognition, Korea Institute of Science and Technology, Seoul, Korea; 2 Department of Chemistry, Kyonggi University, Suwon, Korea; 3 Department of Internal Medicine, Seoul National University College of Medicine, Seoul, Korea

**Keywords:** adrenal tumor, chemical derivatization, mass spectrometry, adrenal cortex, adrenal medullar

## Abstract

Metabolic changes in adrenocortical steroids and medullary catecholamines characterize adrenal tumors, but they are measured using different analytical protocols. To increase bioanalytical validity while maintaining sample homogeneity, LC-MS-based profiling of 29 cortical steroids and 6 medullary amines, including catecholamines and metanephrines, in a single run was developed. Alkyloxycarbonylation with isobutyl chloroformate was employed together with our comprehensive steroid assay, and all adrenal hormones were separated on a reversed-phase C18 column (50 × 2.1 mm, 1.9 μm) at a flow rate of 0.3 ml/min. The lower limits of quantification for all analytes ranged from 0.1 to 2.0 ng/ml, with extraction recoveries of 58.5%–109.5%, while the imprecision and accuracy were 1.6%–14.8% and 89.2%–114.9%, respectively. The validated LC-MS assay was applied to serum samples obtained from 60 patients with adrenal Cushing syndrome, primary aldosteronism, and pheochromocytoma/paraganglioma (PPGL). In addition to the characteristic metabolic changes in glucocorticoids, mineralocorticoids, catecholamines, and metanephrine, the molecular ratios of dehydroepiandrosterone sulfate and 20α-dihydrocortisol indicated Cushing syndrome and primary aldosteronism (*P* < 0.01 for all compounds), respectively. Moreover, the interactive molecular ratios of 11-deoxycortisol with normetanephrine, metanephrine, norepinephrine, and epinephrine (*P* < 0.01 all compounds) were proposed to characterize the metabolic features of PPGL. Novel LC-MS-based quantitative profiling of steroids, catecholamines, and metanephrines in human serum was successfully established and characterized metabolic features of individual adrenal tumors that could be used for clinical purposes.

## Graphical Abstract

**Figure:**
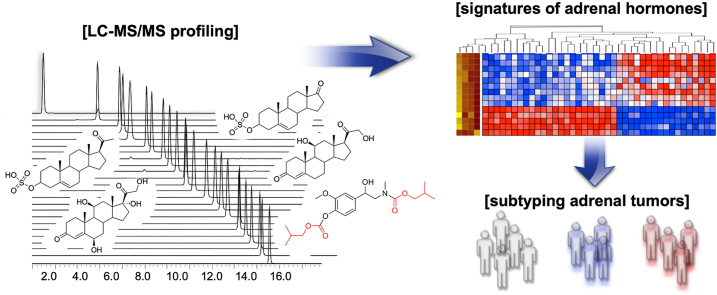

Adrenal glands produce hormones that regulate blood pressure, the immune system, stress response, and reproduction (1). Each gland has an outer cortex, which secretes three distinct classes of steroids, including mineralocorticoids, glucocorticoids, and androgens, whereas the inner medulla produces catecholamines to immediately respond to biochemical stress (2, 3). One common unexpected tumor, adrenal incidentaloma, which is detected by imaging tests, is mostly caused by the hypersecretion of these adrenal cortex and medullary hormones (3, 4).

MS-based multiplexed detection combined with LC-MS or GC-MS could be a promising tool, over the practically used immunoassays, for subtyping adrenal tumors in diagnostic purposes (5, 6, 7). Advanced LC-MS systems can detect various analytes, which have different chemical and physical properties, in a single analytical run based on a high-speed polarity switching technique (8). Although LC-MS profiling of steroids (5, 6, 9, 10) and catecholamines (11, 12) has been widely used in clinical settings to characterize adrenal diseases, steroids and catecholamines are measured in different analytical samplings and runs (13, 14), which may produce biological heterogeneity and clinical diversity.

Because of the different chemical characteristics of adrenal cortical and medullary hormones, a comprehensive method for the simultaneous analysis of both steroids and catecholamines has not been developed for the diagnostic evaluation of adrenal diseases to date. However, the hydrophilic and polar functional groups of amines and catechols were successfully protected by alkyl chloroformates in the aqueous phase with good GC-MS and LC-MS properties (15, 16, 17, 18). To increase the analytical and clinical validity of the screening assays, our steroid assay (9) was modified with an extractable alkyloxycarbonylation of catecholamines and their metabolites, metanephrines.

In this study, we developed and validated simultaneous LC-MS profiles of adrenal cortex steroids, medullary catecholamines, and metanephrines in human serum. This method was then applied to human serum samples obtained from patients with different adrenal tumors, including adrenal Cushing syndrome (CS), primary aldosteronism (PA), and pheochromocytoma/paraganglioma (PPGL), to subtype individual tumors in a single sampling.

## Materials and Methods

### Chemicals

Reference standards for adrenal steroids, catecholamines, and metanephrines (Table 1) were purchased from Sigma-Aldrich (St. Louis, MO) and Steraloids (Newport, RI). Deuterated internal standards (ISs), cortisol-*d*_4_ for 11-deoxycortisol (11-deoxyF), 21-deoxycortisol (21-deoxyF), tetrahydrodeoxycortisol (THS), cortisol, cortisone, 20α-dihydrocortisol (20α-DHF), 6β-hydroxycortisol (6β-OHF), 18-hydroxycortisol (18-OHF), tetrahydrocortisol, allo-tetrahydrocortisol and tetrahydrocortisone (THE); 17α-hydroxyprogesterone-*d*_8_ for 17-hydroxyprogesterone, 21-deoxycorticosterone, corticosterone (B), 18-hydroxycorticosterone (18-OHB), aldosterone and tetrahydroaldosterone; pregnenolone-*d*_4_ for pregnenolone; progesterone-*d*_9_ for progesterone and 11-deoxycorticosterone; 17α-hydroxypregnenolone-*d*_3_ for 17α-hydroxypregnenolone; testosterone-*d*_3_ for androstenedione, 11-hydroxyandrostenedione, testosterone, dihydrotestosterone and 11-hydrotestosterone; dehydroepiandrosterone-*d*_6_ for dehydroepiandrosterone; testosterone sulfate-*d*_3_ for dehydroepiandrosterone sulfate (DHEA-S) and pregnenolone sulfate (P5-S); normetanephrine-*d*_3_ for normetanephrine (NMN); metanephrine-*d*_3_ for metanephrine (MN); 3-methoxytyramine-*d*_4_ for 3-methoxytyramine; norepinephrine-*d*_6_ for norepinephrine (NEN); epinephrine-*d*_6_ for epinephrine (EN); and dopamine-*d*_4_ for dopamine were obtained from C/D/N Isotopes (Pointe-Claire, Quebec, Canada) and Sigma-Aldrich. Sodium acetate (reagent grade), acetic acid (glacial, >99.99%), and formic acid (MS grade, 98%) were purchased from Wako Pure Chemical Industries (Osaka, Japan). Isobutyl chloroformate (>98.0%) and β-glucuronidase extracted from *Escherichia coli* (aqueous solution stabilized with 50% glycerol) were obtained from Tokyo Chemical Industry (Tokyo, Japan) and Roche Diagnostics GmbH (Mannheim, Germany), respectively.

**Table 1:** LC-MS characteristics of steroids, catecholamines, and metanephrines

Compounds (Purity%)	Abbreviation	Scan Mode, Polarity	Molecular Weight (Da)	Precursor ion (*m/z*)	Product ion, *m/z* (CE)^b^	Dwell Weight^c^	Retention Time (min)
Adrenal steroids							
6β-Hydroxycortisol (>98)	6β-OHF	MRM, −	378.2	423.1	347.1 (−22), 312.9 (−42), 205.0 (−42)	1	1.42
18-Hydroxycortisol (>98)	18-OHF	MRM, +	378.2	397.1	267.1 (27), 121.1 (39), 90.9 (87)	1	3.57
20α-Dihydrocortisol (>98)	20α-DHF	MRM, +	364.2	365.1	269.1 (23), 121.0 (33), 91.1 (79)	2	4.49
18-Hydroxycorticosterone (97)	18-OHB	MRM, +	362.2	363.0	121.1 (37), 90.8 (75), 269.1 (22)	2	4.69
Aldosterone (>95)	Aldo	MRM, +	360.2	361.1	343.2 (25), 315.0 (27), 90.9 (81)	2	4.84
Tetrahydroaldosterone (>98)	THAldo	MRM, +	364.5	347.1	329.1 (17), 301.1 (19), 91.1 (75)	2	5.00
Cortisol (>98)	F	MRM, −	362.2	407.0	331.1 (−24), 296.9 (−42), 281.9 (−52)	2	5.58
Cortisone (>98)	E	MRM, −	360.2	405.0	329.1 (−22), 359.1 (−12), 301.0 (−28)	2	5.68
11β-Hydroxytestosterone (>98)	11β-OHT	MRM, +	304.4	305.1	269.3 (23), 121.0 (31), 91.0 (75)	2	6.50
Allo-tetrahydrocortisol (>98)	allo-THF	MRM, −	366.2	411.1	335.3 (−22), 301.1 (−54), 364.8 (−16)	2	6.41
Tetrahydrocortisol (>98)	THF	MRM, −	366.2	411.2	335.2 (−20), 301.1 (−46), 364.9 (−20)	2	6.62
11β-hydroxyandrostenedione (>98)	11-OHA4	MRM, +	302.4	303.1	285.3 (23), 267.1 (25), 121.1 (33)	2	7.16
21-deoxycortisol (>98)	21-deoxyF	MRM, +	346.2	347.1	311.2 (23), 121.0 (31), 105.0 (59)	1.5	7.22
Tetrahydrocortisone (>98)	THE	MRM, −	364.5	409.1	333.3 (−24), 363.1 (−18), 305.2 (−26)	2	7.25
Corticosterone (>98)	B	MRM, +	346.2	347.1	329.2 (21), 121.0 (33), 90.8 (71)	2	7.53
Dehydroepiandrosterone sulfate (>99)	DHEA-S	MRM, −	368.5	367.0	96.9 (−36), 79.9 (−134), 80.4 (−134)	2	7.70
11-Deoxycortisol (>98)	11-deoxyF	MRM, +	346.2	347.1	97.0 (29), 109.1 (33), 79.1 (81)	2	8.02
Testosterone (>98)	T	MRM, +	288.4	289.1	97.1 (27), 109.1 (29), 79.0 (63)	2	10.30
11-Deoxycorticosterone (>98)	DOC	MRM, +	330.2	331.1	97.0 (27), 109.2 (31), 79.0 (79)	2	10.68
21-Deoxycorticosterone (>98)	21-deoxyB	MRM, +	330.2	331.2	313.0 (19), 120.9 (33), 97.0 (33)	2	10.69
Tetrahydrodeoxycortisol (>98)	THS	MRM, +	350.5	351.0	315.2 (15), 297.2 (19), 91.1 (75)	2	10.88
Androstenedione (>98)	A4	MRM, +	286.4	287.1	97.0 (27), 109.1 (29), 79.1 (57)	2	10.90
Pregnenolone sulfate (>98)	P5-S	MRM, −	396.5	395.0	96.9 (−38), 79.8 (−166), 79.1 (−156)	2	10.91
17α-Hydroxypregnenolone (>98)	17α-OHP5	MRM, +	332.5	315.1	297.1 (15), 90.8 (65), 104.8 (55)	2	10.98
Dehydroepiandrosterone (>98)	DHEA	MRM, +	288.4	271.1	253.1 (17), 213.3 (25), 91.0 (53)	2	11.27
17α-Hydroxyprogesterone (>98)	17α-OHP4	MRM, +	330.2	331.1	109.0 (33), 96.9 (31), 79.0 (75)	2	11.44
Dihydrotestosterone (>98)	DHT	MRM, +	290.4	291.1	255.1 (21), 159.1 (29), 90.9 (69)	2	12.05
Progesterone (>98)	P4	MRM, +	314.2	315.1	109.1 (31), 97.1 (25), 79.0 (65)	1	13.40
Pregnenolone (>98)	P5	MRM, +	316.5	317.1	299.3 (13), 281.1 (19), 159.1 (29)	1	13.49
Medullary amines							
Normetanephrine (>98)	NMN	MRM, +	383.2^d^	406.3	288.2 (29), 306.1 (29), 182.1 (29)	2	12.89
Metanephrine (>98)	MN	MRM, +	397.2^d^	420.3	302.2 (31), 320.1 (29), 196.1 (31)	2	13.51
3-Methoxytyramine (>99)	3-MT	MRM, +	367.2^d^	390.3	290.1 (29), 334.1 (27), 173.0 (33)	2	14.00
Norepinephrine (>98)	NEN	MRM, +	469.2^d^	492.3	398.1 (33), 380.3 (35), 280.1 (39)	2	14.42
Epinephrine (>99)	EN	MRM, +	483.2^d^	506.3	406.2 (35), 388.1 (35), 288.1 (37)	2	14.86
Dopamine (>98)	DA	MRM, +	453.2^d^	476.3	376.2 (33), 276.1 (35), 420.2 (29)	2	15.14
Internal standards							
Cortisol-*d*_4_ (+)(98 atom % D)	F-*d*_4_ (+)	MRM, +	366.5	367.1	121.0 (29), 331.1 (23), 92.0 (79)	2	5.57
Cortisol-*d*_4_ (−)(98 atom % D)	F-*d*_4_ (−)	MRM, −	366.5	411.1	331.1 (−24), 296.9 (−42), 281.9 (−52)	2	5.57
Testosterone-*d*_3_ sulfate (98 atom % D)	TS-*d*_3_	MRM, −	371.7	370.0	97.7 (−90), 80.1 (−146), 354 (−56)	2	6.57
Testosterone-*d*_3_ (98 atom % D)	T-*d*_3_	MRM, +	291.4	292.1	109.1 (31), 96.8 (27), 79.2 (67)	2	10.26
17α-Hydroxypregnenolone-*d*_3_ (98 atom % D)	17α-OHP5-*d*_3_	MRM, +	335.5	318.0	300.2 (15), 282.3 (17), 159.2 (29)	2	10.98
Dehydroepiandrosterone-*d*_6_ (98 atom % D)	DHEA-*d*_6_	MRM, +	294.5	295.1	277.2 (11), 259.2 (19), 258.3 (17)	2	11.19
17α-Hydroxyprogesterone-*d*_8_ (98 atom % D)	17α-OHP4-*d*_8_	MRM, +	338.5	339.2	100.2 (29), 321.3 (23), 113.1 (39)	2	11.37
Progesterone-*d*_9_ (98 atom % D)	P4-*d*_9_	MRM, +	323.5	324.2	100.0 (29), 112.9 (31) 85.0 (63)	1	13.35
Pregnenolone-*d*_4_ (98 atom % D)	P5-*d*_4_	MRM, +	320.3	321.2	303.3 (13), 285.1 (17), 159.0 (25)	1	13.46
Normetanephrine-*d*_3_^a^ (97.84)	NMN-*d*_3_	MRM, +	386.2^d^	409.2	290.9 (29), 309.1 (29), 353.2 (27)	2	12.88
Metanephrine-*d*_3_ (98 atom % D)	MN-*d*_3_	MRM, +	400.2^d^	423.3	305.1 (31), 199.2 (33), 323.0 (31)	2	13.49
3-Methoxytyramine-*d*_4_ (98 atom % D)	3-MT-*d*_4_	MRM, +	371.2^d^	394.3	294.1 (29), 338.2 (27), 177.3 (33)	2	13.99
Norepinephrine-*d*_6_ (98 atom % D)	NEN-*d*_6_	MRM, +	475.2^d^	498.3	398.1 (33), 380.3 (35), 280.1 (39)	2	14.40
Epinephrine-*d*_6_ (98 atom % D)	EN-*d*_6_	MRM, +	489.2^d^	512.3	412.3 (35), 394.3 (35), 294.1 (39)	2	14.85
Dopamine-*d*_4_^a^ (96.19)	DA-*d*_4_	MRM, +	457.2^d^	480.3	380.1 (33), 280.2 (35), 424.1 (29)	2	15.13

Quantitative ions are underlined.

a The hydrochloride solution with 100 μg/ml in methanol (as free base) are supplied.

b CE: collision energy.

c Dwell time weighting of scheduled MRM.

d Molecular weights of *N,O*-isobutoxy derivatives.

Oasis HLB cartridges (60 mg, 3 ml) for solid-phase extraction (SPE) was obtained from Waters (Milford, MA), and all organic solvents (analytical and HPLC grade) were obtained from Burdick & Jackson (Muskegon, MI). Strata-X 33-μm polymeric reversed-phase cartridge (60 mg, 3 ml) was supplied by Phenomenex (Milford, MA). Ultrafree-MC centrifugal filter (polyvinylidene fluoride, 0.1 μm pore size) and Amicon 3,000 Da molecular weight cut-off filters were supplied by Millipore (Burlington, MA).

### Standard solutions and surrogated serum samples

Stock solutions of all reference standards were prepared at 1 mg/ml in a mixture of methanol and chloroform (9:1, *v*/*v*). Working solutions were prepared by diluting each stock solution to various concentrations ranging from 0.1 to 1,000 ng/ml for steroids and 1–100 ng/ml for catecholamines and metanephrines. All standard solutions were stored at −20°C until being required.

For calibration purpose, an analyte-free serum was prepared by modifying a commercially available steroid-depleted serum (BBI Solutions; Sittingbourne, UK). In consideration of potential interference encountered with some analytes in the BBI serum (supplemental Fig. S1), additional processing steps were undertaken following our established protocol (8). Briefly, the BBI serum (5 ml) was loaded onto a Strata-X cartridge, subsequently subjected to centrifugation on an Amicon ultrafilter at 12,000 *g* for 20 min. After checking the absence of steroids and medullary amines by LC-MS (supplemental Fig. S2), the stripped serum was employed as the surrogated matrix.

### Instrumental conditions

The LC-MS system consisted of a Shimadzu Nexera ultra-high-performance liquid chromatograph (Shimadzu Corp., Kyoto, Japan) and a triple quadrupole MS combined with a heated electrospray source (QTRAP 6500 plus, AB Sciex, Framingham, MA). All analytes were separated on a reversed-phase Hypersil gold C18 column (50 × 2.1 mm, 1.9 μm particle size; Thermo Fisher Scientific, Waltham, MA) during a 28 min run at a flow rate of 0.3 ml/min. The eluents consisted of 0.1% formic acid in 5% acetonitrile (mobile phase A) and 0.1% formic acid in 95% acetonitrile (mobile phase B). The following gradient was used: 0 min at 10% B; 0–5 min at 10%–25% B; 5–8 min at 25%–30% B; 8–10 min at 30%–35% B; 10–14 min at 35%–70% B; 14–16 min at 70%–100% B (hold 4 min); 20–26 min at 100%–10% B. Subsequently, the initial conditions (10% B) were adopted for 2 min before running the next sample.

The oven temperature was maintained at 40°C, and MS was operated under the following parameters: source temperature, 400°C; curtain gas, 40 au; collisionally activated dissociation gas, medium; ion source gas 1, 55 au; ion source gas 2, 40 au; and ion spray voltage +5500 V and −4500 V for positive and negative ionization, respectively.

For quantification, twenty one of twenty nine steroids were detected in the positive ionization mode, whereas six steroids and two sulfated steroids were detected in the negative ionization mode in the multiple-reaction monitoring mode. All amine derivatives were analyzed using positive ionization in multiple-reaction monitoring mode (Table 1). All peaks were identified by comparing the retention times and matching the height ratios of characteristic ions. Data acquisition was performed using Analyst 1.7 software (AB Sciex; https://sciex.com/products/software/analyst-software).

### Sample pretreatment

**Fig. 1:**
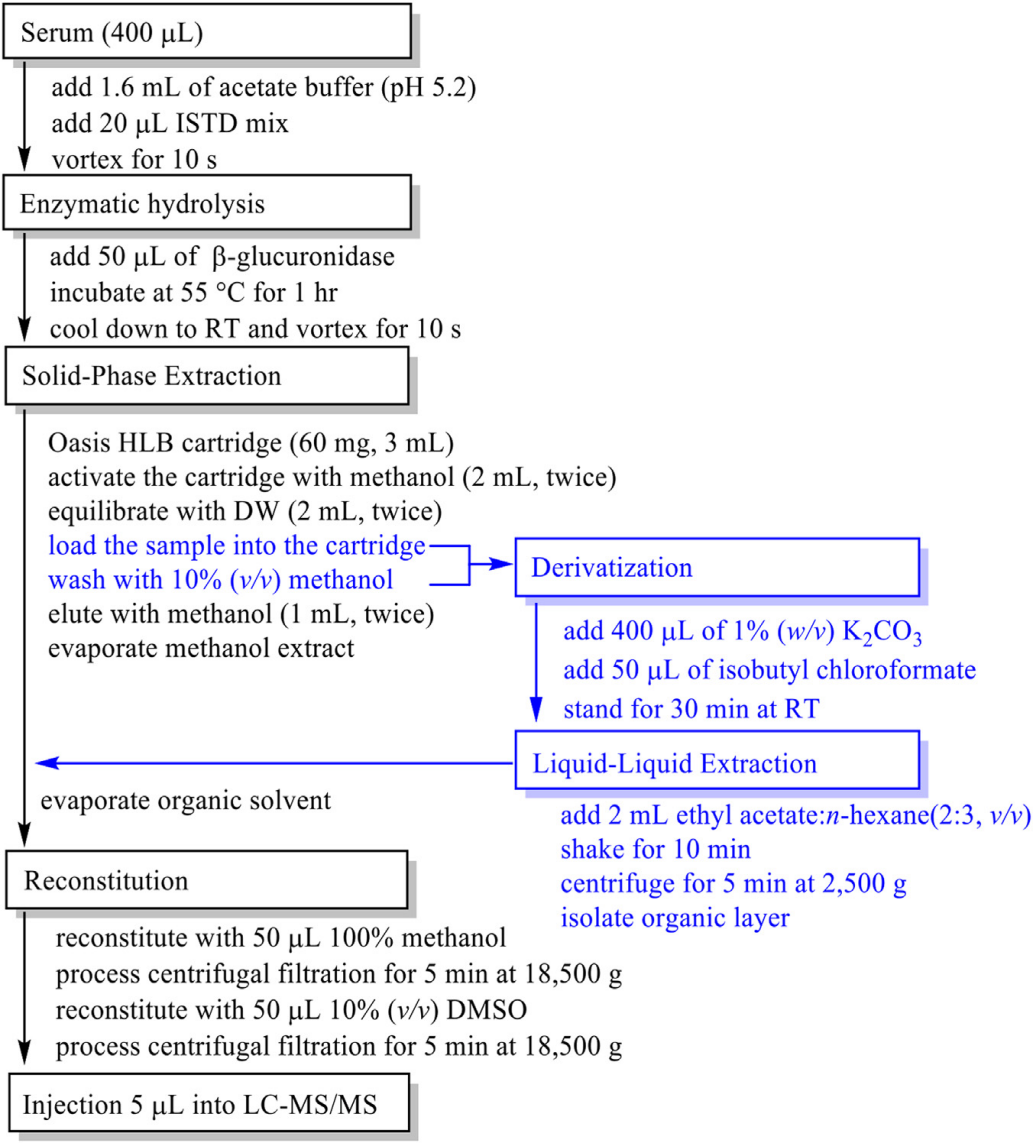
Sample preparation procedures for simultaneous profiling of serum steroids, catecholamines, and metanephrine using LC-MS. The solid-phase extraction, followed by two different sample fractionations of cortical steroids and medullary amines, is processed.

The quantitative profiling assay of adrenal steroids in a previous study (9) was modified for additional detection of catecholamines and metanephrines. Each of the calibrators/quality controls (QCs)/serum samples (400 μl) was diluted with acetate buffer (1.6 ml, pH 5.2) and spiked with 20 μl of internal standard mixtures (cortisol-*d*_4_, 17α-hydroxyprogesterone-*d*_8_, and pregnenolone-*d*_4_: 0.2 μg/ml; progesterone-*d*_9_, 17α-hydroxypregnenolone-*d*_3_, 3-methoxytyramine-*d*_4_, and epinephrine-*d*_6_: 0.1 μg/ml; testosterone-*d*_3_, dopamine-*d*_4_, and norepinephrine-*d*_6_: 0.02 μg/ml; DHEA-*d*_6_: 0.5 μg/ml; testosterone sulfate-*d*_3_: 1 μg/ml; normetanephrine-*d*_3_ and metanephrine-*d*_3_: 0.05 μg/ml). The sample was incubated with 50 μl of β-glucuronidase for 1 h at 55°C. For SPE, an Oasis HLB cartridge (60 mg, 3 ml) was preconditioned with methanol (2 ml, twice), followed by distilled water (2 ml, twice). The hydrolyzed sample was loaded onto a cartridge and washed twice with 10% methanol (0.7 ml). The loading and washing fractions were combined and collected to prepare medullary amines. Steroids were eluted with methanol (1 ml, twice) and evaporated under nitrogen stream at 40°C. The amine fraction was spiked with 400 μl of 1% (*w*/*v*) potassium carbonate and 50 μl of isobutyl chloroformate (*iso*BCF), and then incubated at room temperature for 30 min. After derivatization, the solution was extracted twice with 2 ml of ethyl acetate:*n*-hexane (2:3, *v*/*v*). The organic solvents were combined with the dried residue of the steroid fraction and evaporated under nitrogen stream at 35°C. The dried sample was reconstituted with 50 μl of methanol and centrifuged on an ultrafree-MC centrifugal filter for 5 min at 15,000 g. Thereafter, 50 μl of 10% (*v/v*) dimethyl sulfoxide was added to the ultrafree-MC centrifugal filter and centrifuged for 5 min at 15,000 g. Finally, 5 μl of combined aliquot was injected into the LC-MS system (Fig. 1).

### Method validation

Method validation was performed according to the US Food and Drug Administration 2018 guidelines for bioanalytical method validation (19). The lower limit of quantification (LLOQ) was determined as the concentration at which the signal-to-noise (S/N) ratio was >10. The calibration curve was composed of a double blank (containing only the surrogate), a blank (incorporating both the surrogate and ISs), and a series of eight different concentration samples, ranging from the LLOQ to 100 ng/ml for steroids, except for cortisol and cortisone (200 ng/ml) and DHEA sulfate (1,000 ng/ml), and 20 ng/ml for all medullary amines. The peak height ratios at increasing analyte levels were subjected to least-squares regression analysis to obtain the calibration linearity (*r*^2^) with a 1/*x*^2^ weighting factor. Extraction recovery was established at four different concentrations (LLOQ, low [3- or 5-fold excess of LLOQ], medium, and high concentrations) in the surrogate matrix spiked with all analytes in triplicate. The absolute recoveries were calculated by comparing the peak height ratios of the analytes to the internal standards of the extracted samples versus those of their nonextracted counterparts.

For QC purpose, the pooled human serum was obtained from healthy patients, who had relatively low levels of adrenal hormones. The QC sample with the lowest or low levels of analytes was prepared by diluting 1:3 ratio of a pooled human serum with in-house stripped surrogate serum. QCs with medium and high levels of analytes were prepared by spiking standard working solutions into the pooled serum sample to evaluate imprecision, accuracy, and stability of the method. The concentrations of the QCs were chosen referred to the concentration ranges that are generally measured in the clinic, including the Korean Adrenal Tumor Study (7). The nominal concentration levels of analytes in each QC were sum of the spiked concentration and the mean endogenous level, which was determined by analyzing ten replicate of the unspiked matrix. For intraday imprecision and accuracy, five replicate at each QC concentration were analyzed, while interday imprecision and accuracy were evaluated on three different days. The stability of processed samples under the 24-h storage conditions on (1) auto-sampler (4°C) and (2) room temperature (25°C) was determined using the same QC samples.

### Subjects and sample collection

Patients diagnosed with adrenal incidentaloma at the Department of Internal Medicine, Seoul National University Hospital (Seoul, Korea) were enrolled. CS (aged 56.6 ± 4.2 years, n = 20), PA (55.2 ± 0.7, n = 20) and PPGL (56.1 ± 5.4, n = 20) were diagnosed according to current guidelines (20). Fasting serum samples were collected between 6:00 and 8:00 a.m. and stored at −75°C until analysis. The study was approved by the Institutional Review Board of Seoul National University Hospital (2105-008-1215) and was performed in accordance with the Declaration of Helsinki. Written informed consent was obtained from all the participants.

### Statistical analysis

Data were analyzed using SPSS (version 22; SPSS Inc., Chicago, IL; https://www.ibm.com/support/pages/downloading-ibm-spss-statistics-22) and Prism (version 9.2.0; GraphPad Software Inc., San Diego, CA; https://www.graphpad.com/updates/prism-920-release-notes). The concentrations of steroids and amines in serum samples obtained from the three patient groups were compared using the Kruskal-Wallis test, while the Mann-Whitney test was used to compare groups. Quantitative results and group differences were compared using an unpaired Student’s *t* test. *P*-value < 0.05 was set as significant.

## Results

### Method development

**Fig. 2:**
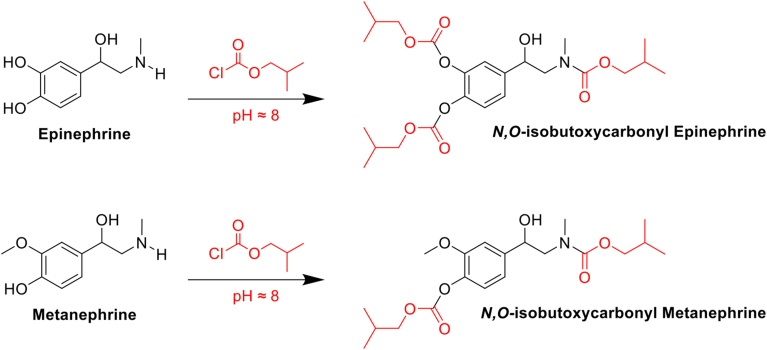
Representative schemes of alkyloxycarbonylation of catecholamine and metanephrine. Both epinephrine and metanephrine are derivatized with isobutyl chloroformate at pH 8 to produce their *N,O*-isobutoxycarbonyl derivatives.

In the initial test, it was necessary to increase the lipophilicity of both catecholamines and metanephrines and chemical derivatization was performed after SPE purification to decrease sample complexity. The derivatizing agent *iso*BCF successfully protected active hydrogens from phenol and amines (Fig. 2) but significantly decreased the extraction recovery of steroids compared to the sample pretreated without the derivatization step (supplemental Fig. S3). Alkyloxycarbonylation with *iso*BCF prior to SPE denatured serum proteins because of the alkaline pH with 1% (*w/v*) potassium carbonate, clogging the SPE cartridge during sample loading. Derivatization after SPE was processed, and both catecholamines and metanephrines were extracted with an excellent recovery in liquid-liquid extraction; however, steroid sulfates, including DHEA-S and P5-S, were not extractable with organic solvents (data not shown).

For reliable analytical efficiency, two sample fractions were prepared for SPE. Catecholamines and metanephrines did not interact with the stationary phase of Oasis HLB during sample loading and were easily washed with the remaining amines in a cartridge with 10% methanol during the washing step. Both fractions were combined and derivatized with *iso*BCF in the aqueous phase and then extracted with a mixture of ethyl acetate:*n*-hexane (2:3, *v*/*v*). In contrast, the extraction fraction with methanol from the SPE, containing steroids, was evaporated, and an amine fraction was added, followed by additional evaporation (Fig. 1). The chromatographic separation of 29 cortical steroids and 6 medullary amines showed a good selectivity and sensitivity through the C18 column within retention times ranging from 1.42 min for 6β-OHF to 15.06 min for dopamine (Table 1).

### Method validation

Method validation for cortical steroids and medullary amines was performed by evaluating the LLOQ, calibration linearity, extraction recovery, imprecision, accuracy, and stability. The LLOQs of all analytes were 0.1–2.0 ng/ml. The devised methods were found to be linear (*r*^2^ > 0.960) over the calibration ranges with the overall extraction recoveries of 58.5%–109.5% (Table 2). Imprecision and accuracy were determined by analyzing QC samples at LLOQ (for NMN and MN) or low, medium, and high concentrations according to the individual sensitivity and calibration range. The overall imprecision (%CV) and accuracy (%bias) ranged from 1.6%–14.8% and 89.2%–114.9%, respectively.

**Table 2:** Method validation in sensitivity, dynamic range, and recovery of the assay

Analyte	LLOQ^a^ (ng/ml)	Calibration Range (ng/ml)	Linearity (*r*^2^)	Recovery (%)
Cortical steroids				
6β-OHF	0.1	0.1–100	0.9900	81.3
18-OHF	0.1	0.1–100	0.9875	102.7
20α-DHF	0.1	0.1–100	0.9924	100.6
18-OHB	0.5	0.5–100	0.9873	109.5
Aldo	2.0	2.0–100	0.9871	79.4
THAldo	0.5	0.5–100	0.9939	82.6
F	0.2	0.2–200	0.9799	95.3
E	0.2	0.2–200	0.9905	89.6
11β-OHT	0.2	0.2–100	0.9981	97.9
allo-THF	1.0	1.0–100	0.9984	89.5
THF	0.5	0.5–100	0.9839	90.8
11-OHA4	0.2	0.2–100	0.9882	94.2
21-deoxyF	0.1	0.1–100	0.9933	94.4
THE	0.2	0.2–100	0.9814	75.6
B	0.2	0.2–100	0.9933	101.9
DHEA-S	1.0	1.0–1000	0.9743	85.9
11-deoxyF	0.1	0.1–100	0.9894	84.7
T	0.1	0.1–100	0.9964	80.7
DOC	0.2	0.2–100	0.9994	82.1
21-deoxyB	0.5	0.5–100	0.9870	88.2
THS	2.0	2.0–100	0.9991	67.2
A4	0.1	0.1–100	0.9923	83.3
P5-S	2.0	2.0–100	0.9726	70.1
17α-OHP5	0.5	0.5–100	0.9987	99.1
DHEA	1.0	1.0–100	0.9744	87.5
17α-OHP4	0.5	0.5–100	0.9985	76.0
DHT	0.2	0.2–100	0.9967	73.0
P4	0.1	0.1–100	0.9983	59.9
P5	1.0	1.0–100	0.9893	58.5
Medullary amines				
NMN	0.2	0.2–20	0.9603	84.9
MN	0.2	0.2–20	0.9973	94.6
3-MT	0.5	0.5–20	0.9729	89.8
NEN	0.5	0.5–20	0.9726	91.4
EN	0.1	0.1–20	0.9925	94.4
DA	0.1	0.1–20	0.9877	90.2

a Lower limit of quantification (LLOQ) was measured according to an S/N ratio >10.

The stability of all adrenal hormones was evaluated using the same QC samples in five replicates. The concentrations of all analytes in the pooled serum samples did not change considerably after being 24 h in auto-sampler (92.8%–114.6% at 4°C) and after exposure to room temperature (86.8%–117.2%, except for 17α-OHP5 of 129.7%, at 25°C) for 24 h (Table 3).

**Table 3:** Method validation in imprecision, accuracy, and stability

Analyte	Nominal Concentration (ng/ml)			Intraday CV (%)			Interday CV (%)			Mean accuracy^a^ (%Bias)			Stability (%)	
	Low	Medium	High	Low	Medium	High	Low	Medium	High	Low	Medium	High	4°C	25°C
Cortical steroids														
6β-OHF	0.3	50.5	75.5	11.7	11.8	7.9	13.4	12.5	11.0	99.3	97.5	103.3	102.1	98.8
18-OHF	0.3	50.3	75.3	14.2	9.4	4.8	11.6	9.8	10.1	105.2	96.5	94.6	114.6	86.8
20α-DHF	0.5	51.4	76.4	11.9	4.8	9.4	12.8	4.9	9.2	106.6	95.7	90.6	113.7	88.6
18-OHB	1.5	52.1	77.1	9.1	3.1	8.4	11.7	8.5	7.7	100.0	93.3	94.3	98.1	94.6
Aldo	5.0	50.0	75.0	4.4	11.0	7.7	12.3	13.3	8.5	100.4	104.1	101.9	109.6	95.1
THAldo	2.0	50.0	75.0	8.2	9.4	5.0	12.7	11.6	5.8	103.5	101.0	100.9	100.3	96.2
F	10.5	92.0	192.0	13.4	10.7	4.8	12.4	12.6	6.3	104.6	101.4	109.9	100.8	99.3
E	3.0	60.1	160.1	11.8	13.0	7.7	13.9	14.8	9.8	103.9	101.8	105.8	103.4	96.3
11β-OHT	0.5	50.0	75.0	12.0	3.5	3.0	13.1	5.9	8.2	105.7	112.8	110.0	106.0	87.7
allo-THF	2.6	52.5	77.5	14.6	9.2	9.1	11.0	12.8	10.4	105.6	98.3	99.7	104.8	100.8
THF	3.4	59.6	84.6	8.6	12.3	8.7	13.1	12.2	10.3	102.2	100.3	100.5	99.3	97.4
11β-OHA4	0.7	50.8	75.8	11.7	7.0	6.1	13.5	8.4	6.7	107.2	111.0	102.1	105.0	89.2
21-deoxyF	0.6	51.8	76.8	9.8	6.5	4.8	10.0	10.1	9.5	109.0	96.4	93.6	108.0	92.5
THE	4.8	67.3	92.3	8.0	12.3	7.1	10.4	13.6	8.7	109.8	107.4	108.7	100.8	100.1
B	0.9	51.8	76.8	12.9	10.7	6.0	11.6	8.9	7.7	102.9	103.8	107.7	106.7	97.6
DHEA-S	27.5	352.2	852.2	5.1	10.3	5.1	7.0	9.5	6.5	112.1	103.8	96.3	98.5	92.6
11-deoxyF	0.3	50.5	75.5	6.1	13.6	4.7	10.8	14.1	5.9	97.1	101.7	107.5	111.1	92.3
T	0.4	50.8	75.8	7.9	6.0	2.5	12.1	9.3	11.0	105.5	102.3	101.8	110.2	89.2
DOC	0.5	50.0	75.0	11.9	9.5	13.2	14.3	8.8	9.2	98.7	101.2	109.3	103.6	88.3
21-deoxyB	1.0	50.0	75.0	7.9	3.1	3.0	7.9	4.9	7.5	102.6	108.9	108.3	102.1	97.3
THS	5.0	50.0	75.0	12.1	13.6	9.1	11.4	12.4	12.3	102.8	105.1	103.6	104.5	95.0
A4	0.3	50.4	75.4	14.3	5.8	2.8	12.9	10.5	11.7	106.3	106.2	103.0	106.7	87.7
P5-S	6.8	57.1	82.1	12.1	4.8	7.5	8.4	13.3	13.6	114.7	101.7	99.1	92.8	96.4
17α-OHP5	5.0	50.0	75.0	11.8	12.0	13.7	12.4	13.6	12.7	101.3	100.8	97.7	93.4	129.7
DHEA	2.0	50.0	75.0	7.8	3.5	3.5	12.7	8.8	10.4	113.8	114.9	111.8	103.3	105.3
17α-OHP4	1.1	50.4	75.4	4.5	4.2	4.6	7.0	3.5	6.5	109.6	106.7	106.4	105.2	95.5
DHT	1.0	50.0	75.0	14.2	8.8	11.8	12.1	7.8	13.1	107.5	104.9	104.1	100.9	88.9
P4	0.6	51.6	76.6	9.0	2.7	1.6	12.0	9.7	8.9	109.7	102.1	104.4	105.1	88.9
P5	5.0	50.0	75.0	3.0	6.7	7.6	13.9	10.0	12.2	103.1	95.3	89.6	101.9	91.6
Medullary amines														
NMN	0.2	10.0	15.0	14.4	8.2	7.0	12.0	13.8	11.6	107.0	101.1	99.3	97.0	97.4
MN	0.2	10.0	15.0	12.6	7.7	8.5	11.0	7.0	6.0	103.6	107.8	108.5	99.5	104.2
3-MT	1.0	10.0	15.0	8.1	6.5	4.6	8.9	10.3	4.9	105.7	103.3	98.8	99.0	97.3
NEN	1.0	10.0	15.0	14.7	11.5	12.0	12.8	12.7	9.8	94.6	103.9	96.4	102.1	117.2
EN	1.0	10.0	15.0	13.5	11.0	3.0	11.7	14.1	12.0	89.2	105.1	100.6	93.6	99.4
DA	1.0	10.0	15.0	12.8	8.5	9.3	13.0	8.7	10.4	98.6	112.3	102.9	96.4	96.9

a The accuracy is expressed as the mean value of data obtained from within intraday and interday runs.

### Application into subtyping adrenal tumors

The validated method was used to measure the serum levels of steroids, catecholamines, and metanephrines in patients with adrenal tumors, including CS, PA, and PPGL. In all subjects, 29 cortical steroids and 6 medullary amines were detected and the quantitative results were compared (supplemental Table S1). The CS group showed elevated levels of glucocorticoids, including 6β-OHF, 20α-DHF, 21-deoxyF, THE, and THS and reduced levels of DHEA and DHEA-S. In contrast, the PA group had increased levels of mineralocorticoids, including B, 18-OHB, and aldosterone, as well as the hybrid steroid 18-OHF. Patients with PPGL had significantly higher levels of all medullary amines.

**Fig. 3:**
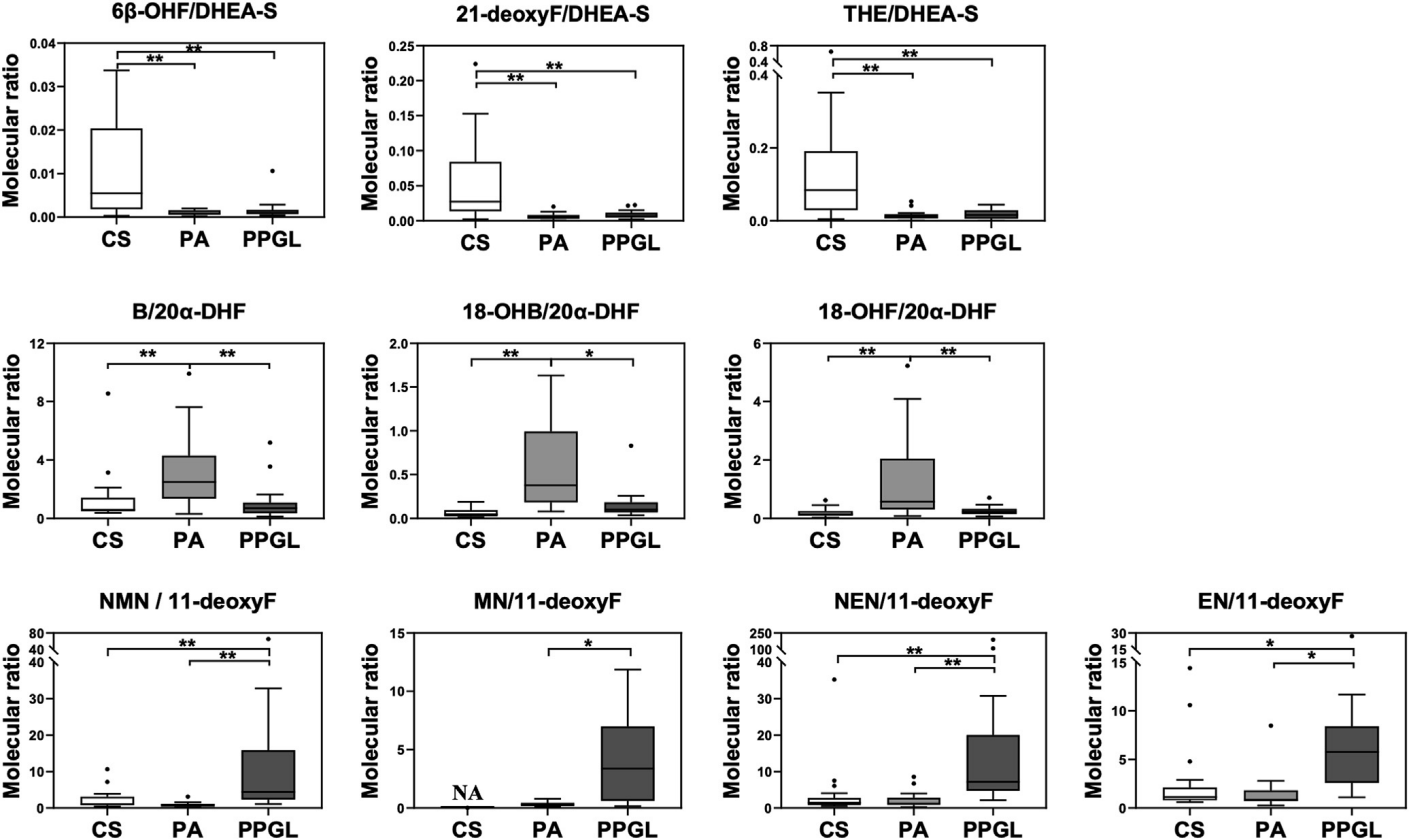
Diagnsotic molecular signatures of serum adrenal hormones. Molecular ratios with dehydroepiandrosterone sulfate (DHEA-S) and 20α-dihydrocortisol (20α-DHF) characterize adrenal Cushing syndrome (CS) and primary aldosteronism (PA), respectively, while four interactive molecular ratios between adrenal cortical and medullary hormones show distinct features of pheochromocytoma/paraganglioma (PPGL). 6β-OHF, 6β-hydroxycortisol; 21-deoxyF, 21-deoxycortisol; B, corticosterone; 18-OHB, 18-hydroxycorticosterone; 18-OHF, 18-hydroxycortisol; EN, epinephrine; MN, metanephrine; NMN, normetanephrine; NEN, norepinephrine; THE, tetrahydrocortisone. ∗*P* < 0.01; ∗∗*P* < 0.001.

In addition to individual adrenal hormones, the metabolic ratios of DHEA-S and 20α-DHF also effectively characterized CS and PA, respectively, from corresponding adrenal tumors (*P* < 0.01). The interactive molecular ratios between the cortex and medullary hormones, such as NMN/11-deoxyF (*P* < 0.001), MN/11-deoxyF (*P* < 0.01), NEN/11-deoxyF (*P* < 0.001), and EN/11-deoxyF (*P* < 0.01), successfully differentiated PPGL from both CS and PA in this study (Fig. 3).

## Discussion

In contrast to MS-based serum profiling of cortical steroids and medullary amines which are carried out in different analytical runs (13, 14), this study aimed to develop a novel LC-MS method for the profiling of adrenal hormones, including steroids, catecholamines, and metanephrines, in a single analytical run to maintain the sample homogeneity and bioanalytical validity. Catecholamines and metanephrines have different chemical properties from steroids, which have a lipophilic 4-membered hydrocarbon ring system. Therefore, it is an analytical challenge to simultaneously quantify hydrophilic catechol/side-chain amines with steroids.

Amines were derivatized to improve analytical efficiency because of their proton affinity and uneven distribution of their electrons, which could reduce chromatographic separation in reversed-phase LC-MS analysis (21). Alkyl chloroformates react immediately with primary and secondary amines in the aqueous phase (15, 16, 18, 21). In this study, derivatization was performed using *iso*BCF at room temperature resulting in an excellent peak shape and signal-to-noise ratio (supplemental Fig. S4).

The established method reflects the clinical signatures of individual adrenal tumors of CS (5, 13, 22, 23), PA (5, 13,1 4)), and PPGL (11, 12, 14) characterized by excessive secretion of glucocorticoids, mineralocorticoids, and catecholamines, respectively. A recent study suggested the concept of adrenal cortical-medullary interaction involving the influence of catecholamines on adrenal steroids in patients with PPGL (13). However, cross-talk between the adrenal cortex and medulla has not yet been clearly investigated, and interactive molecular signatures would be better estimated from the sample.

In conclusion, a comprehensive LC-MS-based profiling method was developed to simultaneously quantify serum steroids, catecholamines, and metanephrine in a single analytical run. The validated assay was used to identify the metabolic signatures of individual adrenal tumors such as CS, PA, and PPGL, and it successfully identified well-known molecular biomarkers. In addition, this novel method could suggest interactive molecular signatures between the cortex and medulla of the adrenal gland in different adrenal tumors, which could be used in clinical settings to subtype adrenal tumors. In further studies, the metabolic roles of DHEA-S, 20α-DHF, and 11-deoxyF associated with adrenal tumors would be investigated.

## Data availablility

The data supporting this study are available in the article, the supplemental data, or available from the corresponding author upon reasonable request.

## Supplemental data

This article contains supplemental data.

## Conflict of interest

The authors declare that they have no conflicts of interest with the contents of this article.
