# Supplementary material for: LC-MS based simultaneous profiling of adrenal hormones of steroids, catecholamines, and metanephrines
Source: J Lipid Res. 2023 Oct 6;64(11):100453. doi: 10.1016/j.jlr.2023.100453 (PMC10641533; doi:10.1016/j.jlr.2023.100453)
Supplement: Supplemental data [file mmc1.pdf]

*Supplemental data*

# LC-MS based simultaneous profiling of adrenal hormones of steroids, catecholamines, and metanephrines

**Jongsung Noh[1,2,a], Chaelin Lee[1,a], Jung Hee Kim[3], Seung Woon Myung[2], and Man Ho Choi[1,*]**

[1]Center for Advanced Biomolecular Recognition, Korea Institute of Science and Technology, Seoul 02792, Korea; [2]Department of Chemistry, Kyonggi University, Suwon 16227, Korea; [3]Department of Internal Medicine, Seoul National University College of Medicine, Seoul 03080, Korea

[a]The authors contributed equally to this study.
*To whom correspondence should be addressed: mh_choi@kist.re.kr

Number of figures: 4
Number of tables: 1

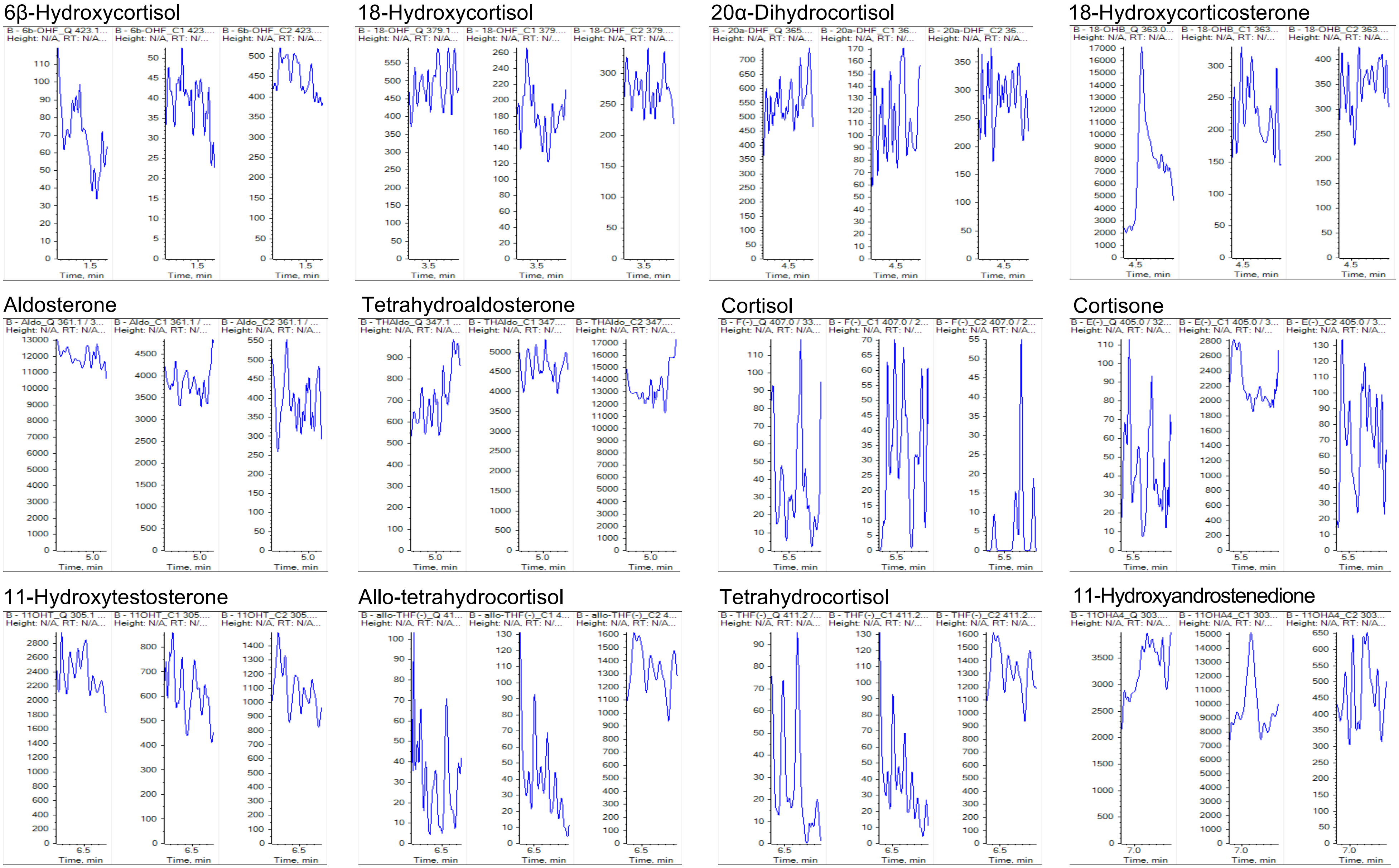


**Supplemental Fig. S1. Chromatographic profiles of 29 steroids and 6 amines in the steroid-depleted serum.** The quantitative ion (left), the first qualitative ion (middle), and the second qualitative ion (right) of individual analytes were monitored. All characteristic ions are listed in Table 1.

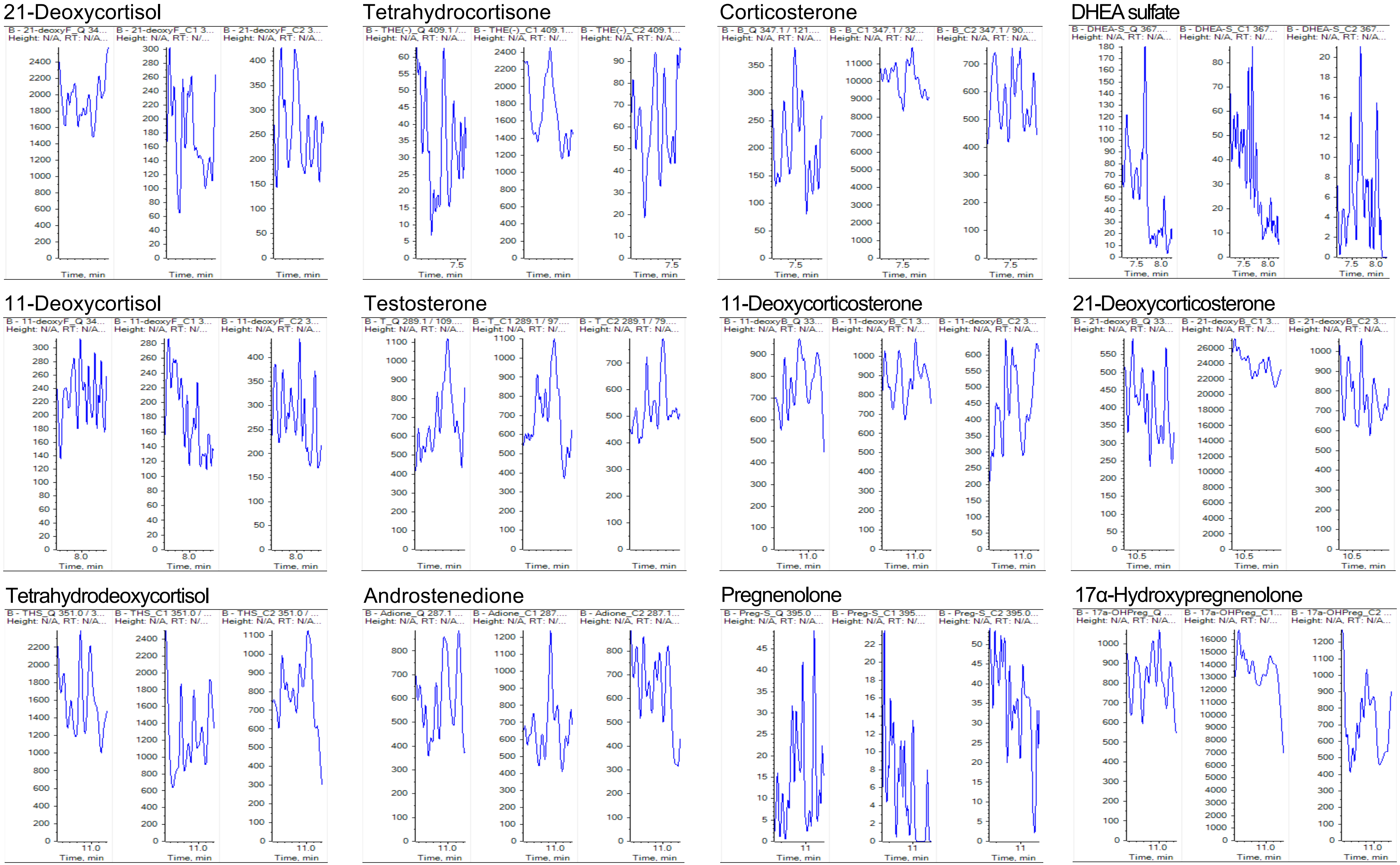


**Supplemental Fig. S1.** Continued.

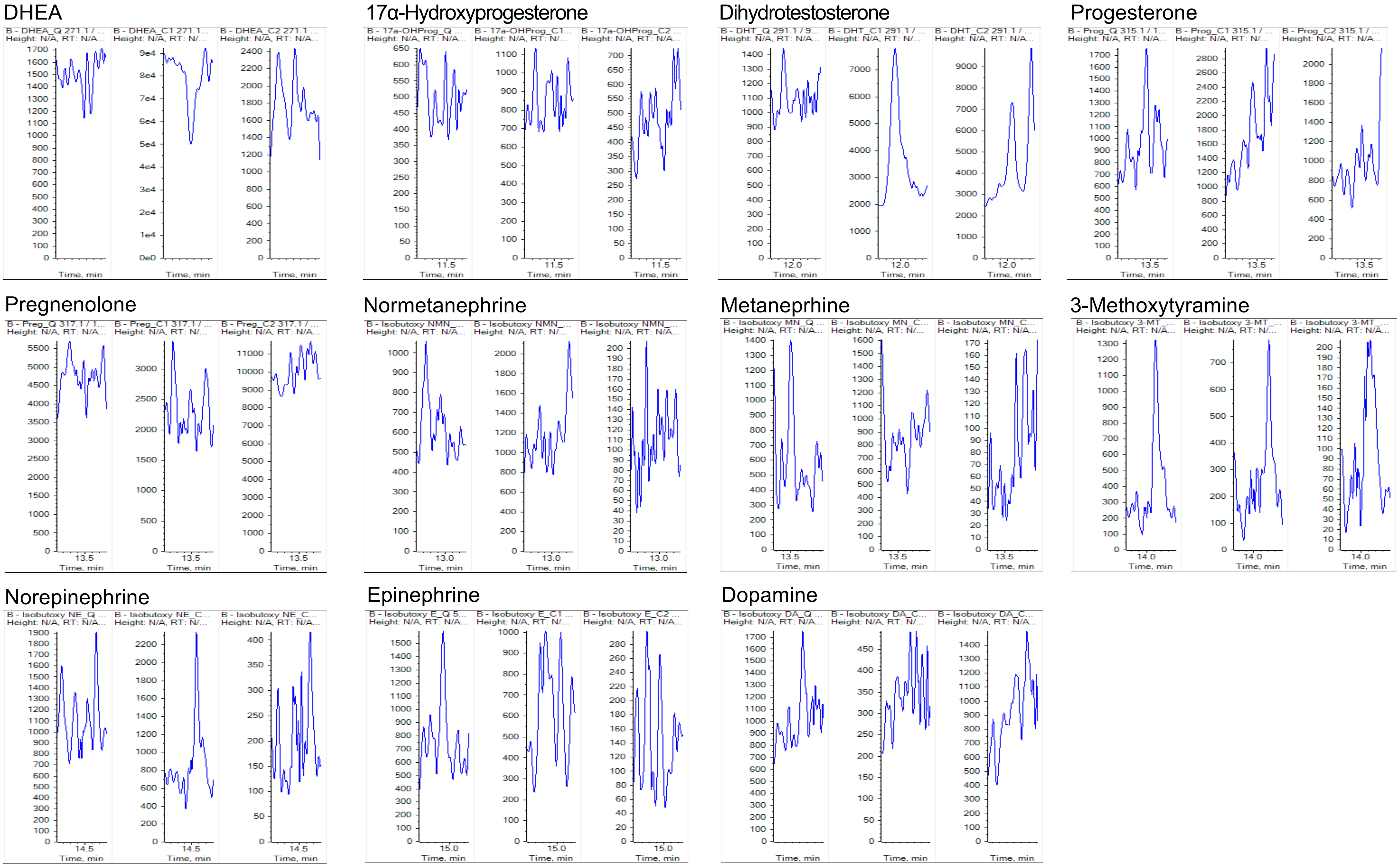


**Supplemental Fig. S1.** Continued.

## [Cortical Steroids]

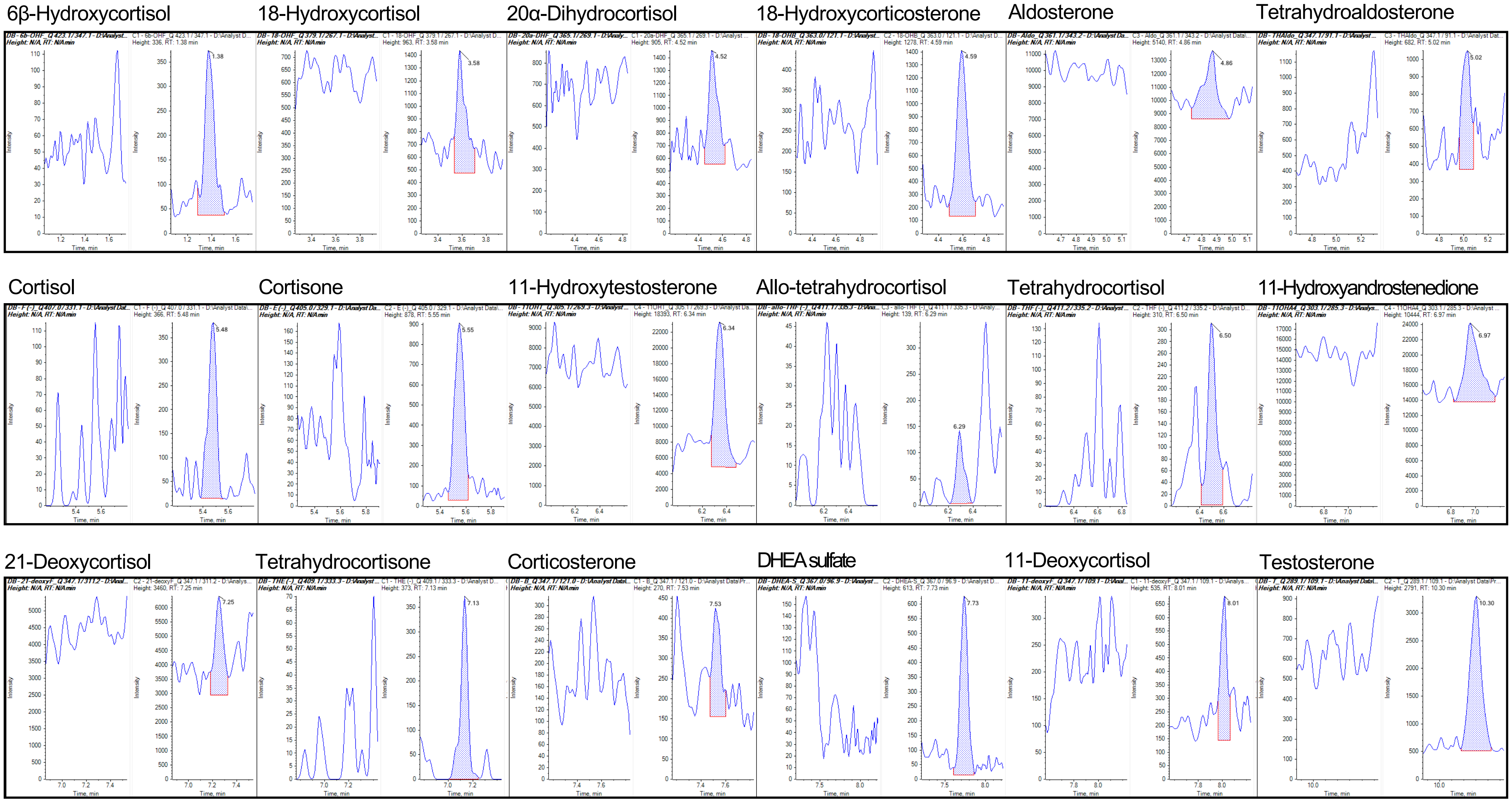


**Supplemental Fig. S2. Chromatographic profiles of 29 steroids and 6 amines in the stripped surrogate serum.** Chromatograms are obtained from a double blank (left) and a compound spiked at LLOQ level of individual analytes. All internal standards were spiked at the levels of used in the assay.

11-Deoxycorticosterone | 21-Deoxycorticosterone | Tetrahydrodeoxycortisol | Androstenedione | Pregnenolone sulfate | 17α-Hydroxypregnenolone

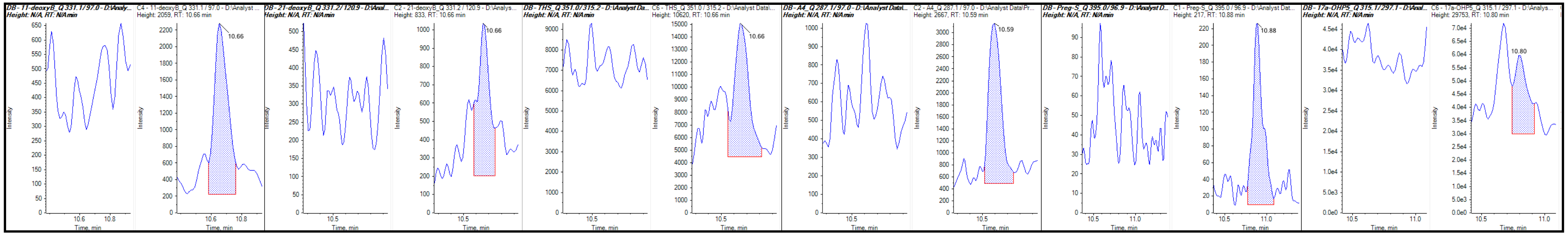

DHEA | 17α-Hydroxyprogesterone | Dihydrotestosterone | Progesterone | Pregnenolone

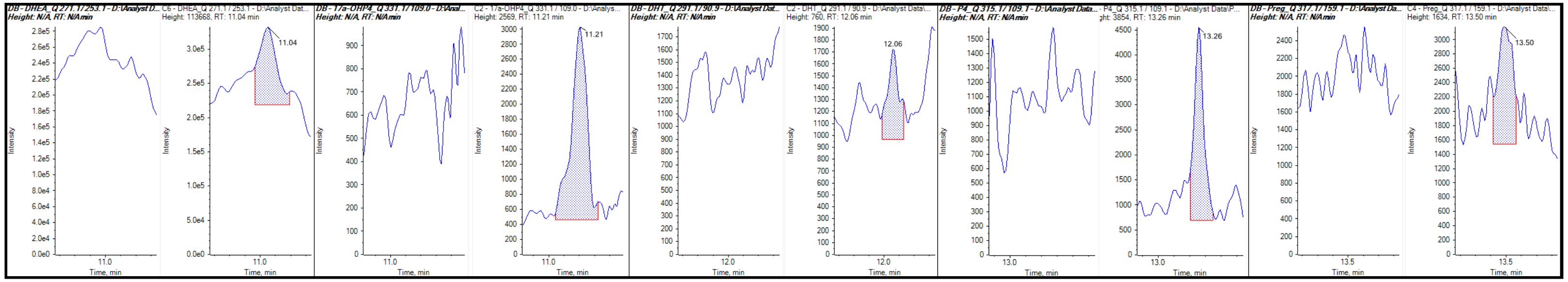

## [Medullary amines]

Normetanephrine | Metaneprhine | 3-Methoxytyramine | Norepinephrine | Epinephrine | Dopamine

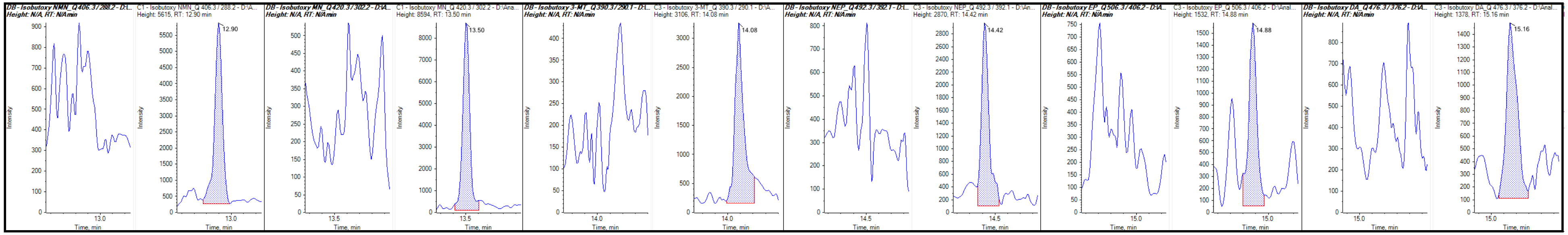

**Supplemental Fig. S2.** Continued.

## [Internal standards for cortical steroids]

$d_4$-Cortisol (+) $d_4$-Cortisol (-) $d_3$-Testosterone sulfate $d_3$-Testosterone $d_3$-17α-Hydroxypregnenolone $d_6$-DHEA

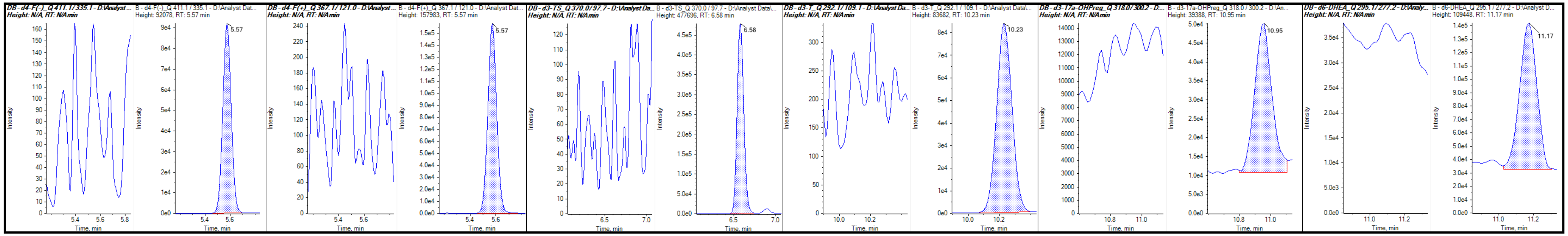

$d_8$-17α-Hydroxyprogesterone $d_9$-Progesterone $d_4$-Pregnenolone

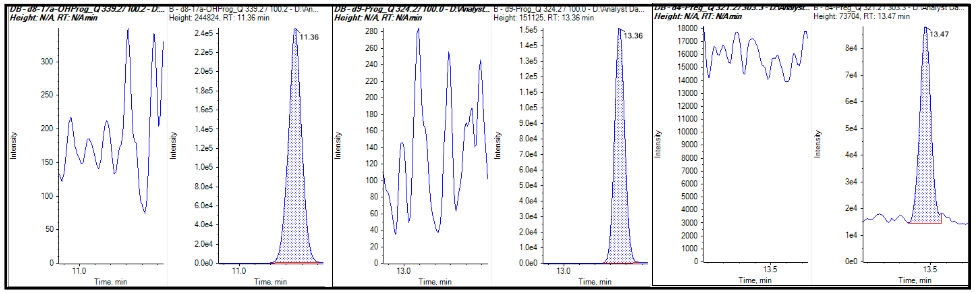

## [Internal standards for medullary amines]

$d_3$-Normetanephrine $d_3$-Metanephrine $d_4$-3-Methoxytyramine $d_6$-Norepinephrine $d_6$-Epinephrine $d_4$-Dopamine

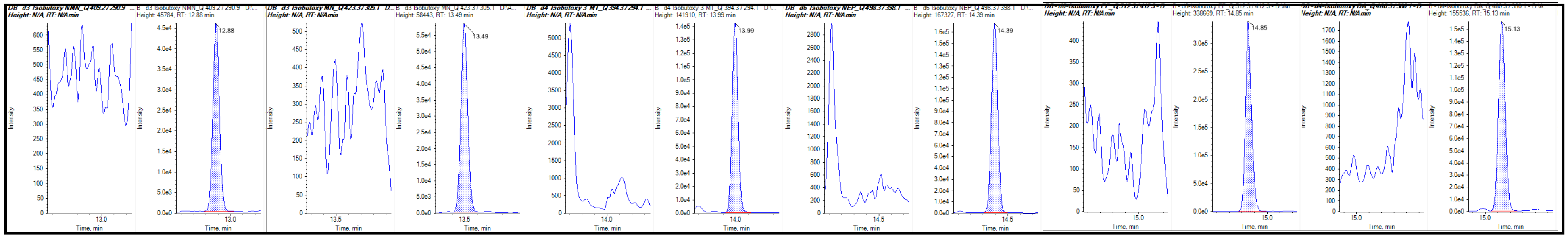

**Supplemental Fig. S2.** Continued.

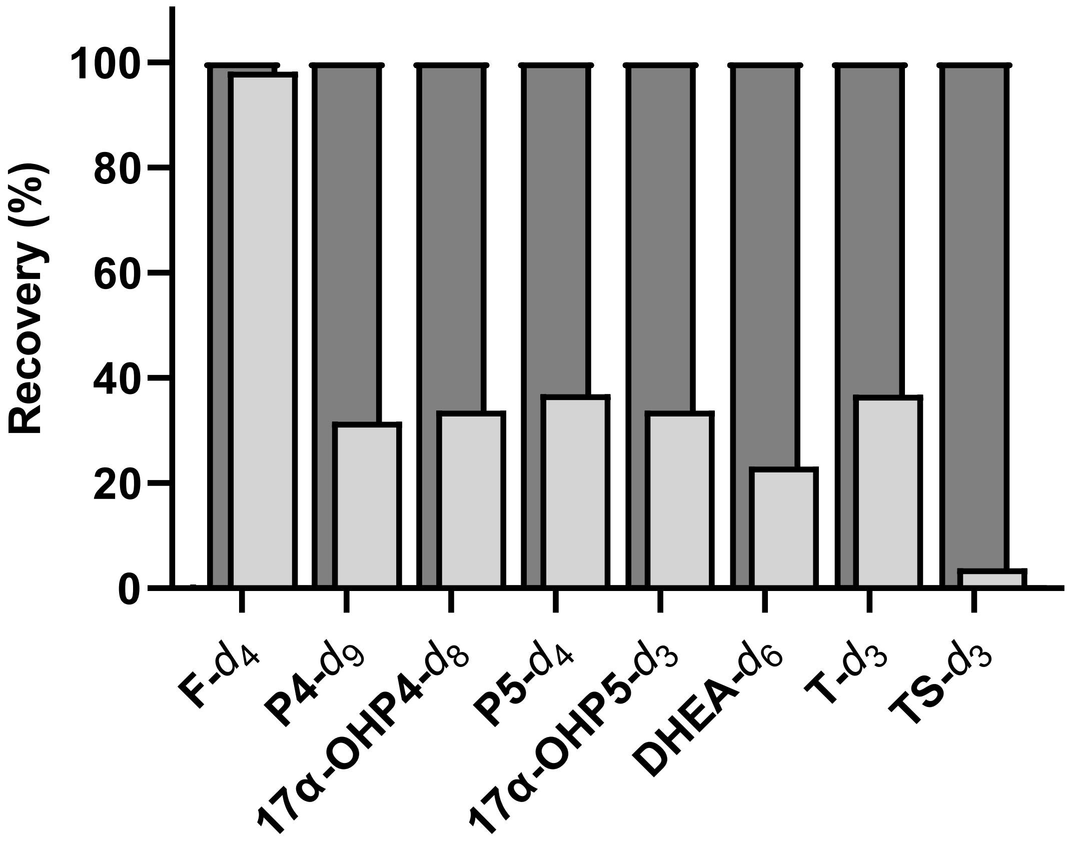


**Supplemental Fig. S3. Comparative extraction efficacy of steroids affected by alkyloxycarbonylation.** Eight different steroids corresponding their deuterium labeled internal standards were tested, and extraction recovery of all steroids significantly declined (3.8–36.9%), except for cortisol (98.3%), compared with those from the extraction protocol without the derivatization step. F, cortisol; P4, progesterone; 17α-OHP4, 17α-hydroxyprogesterone; P5, pregnenolone; 17α-OHP5, 17α-hydroxypregnenolone; DHEA, dehydroepiandrosterone; T, testosterone, TS, testosterone sulfate.

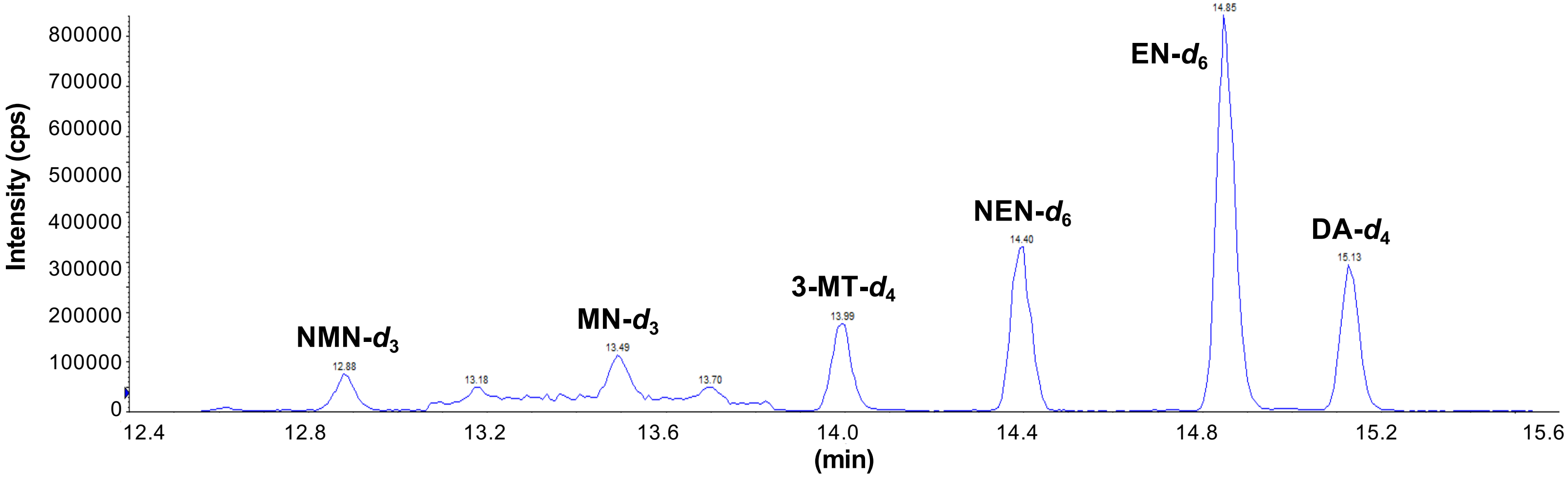


**Supplemental Fig. S4. A representative chromatogram of catecholamines and metanephrines in the serum sample.** All internal standards show a good chromatographic separation. NMN-$d_3$, normetanephrine-$d_3$; MN-$d_3$, metanephrine-$d_3$; 3-MT-$d_4$, 3-methoxytyramine-$d_4$; NEN-$d_6$, norepinephrine-$d_6$; EN-$d_6$, epinephrine-$d_6$; DA-$d_4$, dopamine-$d_4$.

**Supplemental Table 1.** Comparative serum levels (ng/mL) of adrenal steroids, catecholamines, and metanephrines between patient groups

| Compounds | CS | PA | PPGL | *P*-value | | |
|---|---|---|---|---|---|---|
| | Mean ± SD | Mean ± SD | Mean ± SD | CS vs PA | PA vs PPGL | CS vs PPGL |
| ***Cortical steroids*** | | | | | | |
| 6β-OHF | 1.48 ± 0.84 | 0.67 ± 0.30 | 0.83 ± 0.70 | **< 0.001** | 0.695 | **0.002** |
| 18-OHF | 0.45 ± 0.30 | 1.48 ± 1.52 | 0.49 ± 0.29 | **0.003** | **0.003** | 0.579 |
| 20α-DHF | 2.99 ± 2.20 | 1.54 ± 0.83 | 2.13 ± 1.18 | **0.022** | 0.096 | 0.337 |
| F | 118.27 ± 69.64 | 93.79 ± 45.14 | 81.40 ± 38.21 | 0.482 | 0.304 | 0.099 |
| E | 21.11 ± 5.44 | 17.15 ± 7.56 | 18.50 ± 7.88 | 0.123 | 0.626 | 0.317 |
| allo-THF | 21.35 ± 21.97 | 19.54 ± 11.57 | 14.58 ± 8.13 | 0.579 | 0.152 | 0.330 |
| THF | 29.59 ± 19.36 | 19.27 ± 9.99 | 21.07 ± 12.28 | 0.088 | 0.871 | 0.185 |
| 21-deoxyF | 7.16 ± 2.80 | 4.02 ± 2.15 | 4.55 ± 1.96 | **< 0.001** | 0.588 | **0.002** |
| THE | 20.95 ± 13.79 | 9.32 ± 5.11 | 9.32 ± 5.65 | **< 0.001** | 0.892 | **< 0.001** |
| 11-deoxyF | 0.58 ± 0.33 | 0.92 ± 0.66 | 0.31 ± 0.23 | 0.079 | **< 0.001** | **0.008** |
| THS | 6.51 ± 4.32 | 4.18 ± 2.34 | 3.14 ± 3.12 | 0.123 | 0.172 | **0.006** |
| 18-OHB | 0.19 ± 0.12 | 0.73 ± 0.47 | 0.33 ± 0.41 | **< 0.001** | **0.005** | 0.213 |
| Aldo | 0.19 ± 0.04 | 0.38 ± 0.22 | 0.29 ± 0.25 | **0.021** | **0.006** | 0.220 |
| THAldo | 0.97 ± 0.30 | 1.28 ± 0.76 | 1.40 ± 0.98 | **0.029** | 0.369 | 0.376 |
| B | 2.84 ± 2.21 | 4.55 ± 3.35 | 2.29 ± 2.59 | **0.048** | **0.014** | 0.204 |
| DOC | 0.14 ± 0.14 | 0.52 ± 0.43 | 0.47 ± 0.25 | 0.288 | 0.236 | 0.709 |
| 21-deoxyB | 0.21 ± 0.11 | 0.32 ± 0.20 | 0.30 ± 0.23 | 0.118 | **0.003** | 0.083 |
| P5-S | 31.74 ± 41.39 | 23.07 ± 12.80 | 23.89 ± 13.24 | 0.358 | 0.978 | 0.304 |

| | | | | | | |
|---|---|---|---|---|---|---|
| 17α-OHP5 | 3.51 ± 2.59 | 3.69 ± 1.18 | 2.30 ± 1.20 | **0.039** | 0.152 | 0.287 |
| 17α-OHP4 | 0.36 ± 0.37 | 0.68 ± 0.54 | 0.49 ± 0.39 | **0.041** | 0.250 | 0.245 |
| P4 | 0.06 ± 0.03 | 0.08 ± 0.05 | 0.11 ± 0.10 | 0.140 | 0.050 | 0.539 |
| P5 | 0.60 ± 0.22 | 0.64 ± 0.26 | 0.74 ± 0.35 | **0.040** | 0.253 | 0.405 |
| 11β-OHT | 0.19 ± 0.12 | 0.19 ± 0.17 | 0.20 ± 0.14 | 0.817 | 0.503 | 0.639 |
| 11-OHA4 | 1.82 ± 1.29 | 2.78 ± 1.71 | 1.74 ± 1.05 | 0.133 | 0.064 | 0.745 |
| DHEA-S | 670.75 ± 980.05 | 816.58 ± 386.09 | 687.50 ± 317.14 | **0.011** | 0.482 | **0.030** |
| T | 1.60 ± 2.67 | 2.44 ± 2.59 | 2.52 ± 2.79 | 0.457 | 0.787 | 0.234 |
| A4 | 0.46 ± 0.25 | 0.95 ± 0.51 | 0.60 ± 0.28 | **0.017** | 0.126 | 0.102 |
| DHEA | 1.25 ± 0.94 | 2.92 ± 1.70 | 2.94 ± 1.77 | **< 0.001** | 0.808 | **< 0.001** |
| DHT | 0.40 ± 0.15 | 0.33 ± 0.18 | 0.48 ± 0.51 | 0.200 | 0.807 | 0.318 |
| ***Medullary amines*** | | | | | | |
| NMN | 0.62 ± 0.16 | 0.52 ± 0.18 | 1.54 ± 0.91 | 0.319 | **< 0.001** | **< 0.001** |
| MN | ND[a] | 0.10 ± 0.03 | 0.88 ± 1.15 | NA[b] | **0.007** | NA[b] |
| 3-MT | ND[a] | 0.92 ± 1.37 | 3.40 ± 2.72 | NA[b] | 0.338 | NA[b] |
| NEN | 0.86 ± 0.34 | 1.02 ± 0.35 | 3.36 ± 3.68 | 0.123 | **< 0.001** | **< 0.001** |
| EN | 0.69 ± 0.22 | 0.85 ± 0.24 | 1.31 ± 0.92 | **0.030** | 0.745 | 0.116 |
| DA | 0.49 ± 0.17 | ND[a] | 0.60 ± 0.17 | NA[b] | NA[b] | **0.007** |

[a] Not detected.
[b] Not acceptable.
